# Clinical features and brain structural changes in magnetic resonance imaging in Alzheimer’s disease patients with apathy

**DOI:** 10.18632/aging.103705

**Published:** 2020-10-11

**Authors:** Shu-Yang Yu, Wan-Lin Zhu, Peng Guo, Shao-Wu Li, Ya-Ou Liu, Teng-Hong Lian, Du-Yu Ding, Dan-Ning Li, Li-Xia Li, Li Liu, Hui Zhao, Li-Jun Zuo, Yang Hu, Qiu-Jin Yu, Zhao Jin, Rui-Dan Wang, Jun-Hua Gao, Rong-Yan Zhu, Xiao-Min Wang, Wei Zhang

**Affiliations:** 1 Department of Neurology, Beijing Tiantan Hospital, Capital Medical University, Beijing 100070, China; 2 China National Clinical Research Center for Neurological Disease, Beijing Tiantan Hospital, Capital Medical University, Beijing 100070, China; 3 Beijing Neurosurgical Institute, Beijing 100070, China; 4 Department of Radiology, Beijing Tiantan Hospital, Capital Medical University, Beijing 100070, China; 5 Department of General Internal Medicine, Beijing Tiantan Hospital, Capital Medical University, Beijing 100070, China; 6 Department of Physiology, Capital Medical University, Beijing 100069, China; 7 Center for Cognitive Neurology, Department of Neurology, Beijing Tiantan Hospital, Capital Medical University, Beijing 100070, China; 8 China National Clinical Research Center for Neurological Disease, Beijing Tiantan Hospital, Capital Medical University, Beijing 100070, China;; 9 Center of Parkinson's Disease, Beijing Institute for Brain Disorders, Beijing 100053, China; 10 Beijing Key Laboratory on Parkinson's Disease, Beijing 100053, China

**Keywords:** Alzheimer’s disease, apathy, clinical features, brain structural changes, magnetic resonance imaging

## Abstract

Background: Apathy is common in Alzheimer’s disease (AD) patients. However, its relation with other clinical symptoms in AD and brain structural changes in magnetic resonance imaging is unclear.

Results: Compared with AD with no apathy group, cognitive function and activities of daily living were significantly impaired and neuropsychiatric symptoms were obviously presented in AD with apathy group (P<0.05). The frequency of Apolipoprotein E genotypes was not significantly different (P>0.05). Correlation analyses and multiple linear analyses revealed that thickness of left temporal pole and volume of posterior corpus callosum were significantly and negatively correlated with Modified Apathy Estimation Scale score in AD patients (P<0.05).

Conclusions: Apathy with AD is positively correlated with cognitive impairment, neuropsychiatric symptoms and poor activities of daily living. Atrophy of left temporal pole and posterior corpus callosum presented by MRI is positively related with apathy of AD.

Methods: In this study, 137 AD patients were recruited and divided into AD with apathy group and AD with no apathy group according to Modified Apathy Estimation Scale score. We evaluated patients’ cognitive function, neuropsychiatric symptoms and activities of daily living, detected the frequency of Apolipoprotein E genotypes and measured cortical thickness and volume by magnetic resonance imaging (MRI).

## INTRODUCTION

Apathy is characterized by the loss of or diminished motivation in at least two out of three domains: goal-directed behavior, cognitive activity and emotion. Apathy is the most common neuropsychiatric symptom in patients with Alzheimer’s disease (AD). Based on the score of apathy in Neuropsychiatric Inventory (NPI), a group reported the prevalence of apathy to be 2% in cognitively normal persons, 39% in mild cognitive impairment (MCI) due to AD, and 51% in mild AD [1]. The same group also reported a 1-month prevalence of 72% among 50 outpatients with mild to severe AD, which demonstrated an increased prevalence of apathy as dementia severity worsened [2]. Apathy has been proposed to be a signal of imminent cognitive decline [3, 4] and future risk for dementia in patients with AD [5]. Thus, clinicians should pay great attention to this easily ignored neuropsychiatric symptom in AD. Neuropsychiatric symptoms in AD, such as apathy, depression, aggression, agitation, sleep disruption and psychosis were recognized as the important manifestations of AD, which were expressed to varying degrees throughout the course of AD [6]. Apathy often coexists with other neuropsychiatric symptoms, thus, they may share some pathogenic mechanisms. However, the unique pathogenic process of apathy and its relation to other neuropsychiatric symptoms still remain unknown.

Brain structural studies by magnetic resonance imaging (MRI) in AD patients reported an association between apathy and abnormalities in the anterior cingulate cortex and its connections with the orbitofrontal cortex and the basal ganglia [7]. From the anterior cingulate cortex, efferent fibers project to the head of the caudate, substantia nigra and ventral anterior nucleus of thalamus, then the circuit is closed by projections from the thalamus back to the anterior cingulate cortex. The anterior cingulate cortex is considered as the key structure of this circuit. However, damage in other brain areas, such as subcortical and brainstem, etc., is also reported to be related to apathy. In a word, apathy is an important symptom of AD, and studies about the relation between apathy and brain structural changes in MRI are few and the results are not consistent yet.

## RESULTS

In this study, 66 of 137 (48.2%) AD patients had apathy. Comparison of demographics information between AD-A and AD-NA groups showed that gender, age, disease duration and educational level were not significantly different (P>0.05) (Table 1). Correlation analyses suggested that MAES score was not associated with age and disease duration (P>0.05).

**Table 1:** Demographic variables of AD-A^1^ and AD-NA^2^ groups.

**Demographic variables**	**AD-A group (66 cases)**	**AD-NA group (71 cases)**	**(P value)**	**Coefficient P value**
Gender			0.889	
Male [case (%)]	24 (36.4%)	28 (39.4%)		
Female [case (%)]	42 (63.6%)	43(60.6%)		
Age [year, median (quartile)]	66.5(59.0~77.25)	67.5 (60.0~76.3)	0.510	-0.021(0.811)
Disease duration [year, median (quartile)]	3.0(2.0~5.0)	2.0 (1.5~4.3)	0.115	0.133(0.128)
Educational level			0.294	
Illiteracy [case (%)]	4 (6.1%)	2 (2.8%)		
Primary school [case (%)]	13 (19.7%)	9 (12.7%)		
Middle school [case (%)]	15 (22.7%)	18 (25.4%)		
High school [case (%)]	22 (33.3%)	18 (25.4%)		
College and above [case (%)]	11(16.7%)	23 (32.4%)		
MMSE^3^ [scores, median (quartile)]	18.0(9.5~25.0)	26.0(23.0~29.0)	0.000*	-0.527(0.000*)
NPI^4^[scores, median (quartile)]	5.5 (1.3~19.8)	1.0 (0~3.0)	0.000*	0.468(0.000*)
ADL^5^[scores, median (quartile)]	27.0(20.0~54.0)	20.0(20.0~24.0)	0.000*	0.443(0.000*)

*P<0.007 after Bonferroni correction. ^1^AD-A: Alzheimer’s disease with apathy, ^2^AD-NA: Alzheimer’s disease with no apathy,^3^ MMSE: Mini-Mental State Examination, ^4^ NPI: Neuropsychiatric Symptom Inventory, ^5^ ADL: Activities of Daily Living.

Compared with AD-NA group, the score of MMSE scale was significantly decreased and the scores of NPI and ADL scales were significantly increased in AD-A group (P<0.05). Correlation analyses demonstrated that the MAES score was significantly and negatively correlated with the score of MMSE scale, and the scores of NPI and ADL scales were significantly and positively correlated with the score of MMSE scale (P<0.05) (Table 1).

The frequency of ApoE genotypes, including ε2/ ε2, ε2/ ε3, ε2/ ε4, ε3/ ε3, ε3/ ε4, and ε4/ ε4, was not significantly different between AD-A and AD-NA groups (P>0.05) (Table 2).

**Table 2:** The frequencies of ApoE^1^ genotypes of AD-A^2^ and AD-NA^3^ groups.

**ApoE genotypes**	**AD-A group (33 cases)**	**AD-NA group (37 cases)**	**P value**
ε2/ ε2[case (%)]	0 (0%)	0 (0%)	0.602
ε2/ ε3[case (%)]	3(9.0%)	3(8.1%)	
ε2/ ε4[case (%)]	0 (0%)	1(2.7%)	
ε3/ ε3[case (%)]	20(60.6%)	26(78.8%)	
ε3/ ε4[case (%)]	9(27.3%)	7(18.9%)	
ε4/ ε4[case (%)]	1(3.0%)	0 (0%)	

^1^ ApoE: Apolipoprotein E, ^2^AD-A: Alzheimer’s disease with apathy, ^3^AD-NA: Alzheimer’s disease with no apathy.

**Figure 1:**
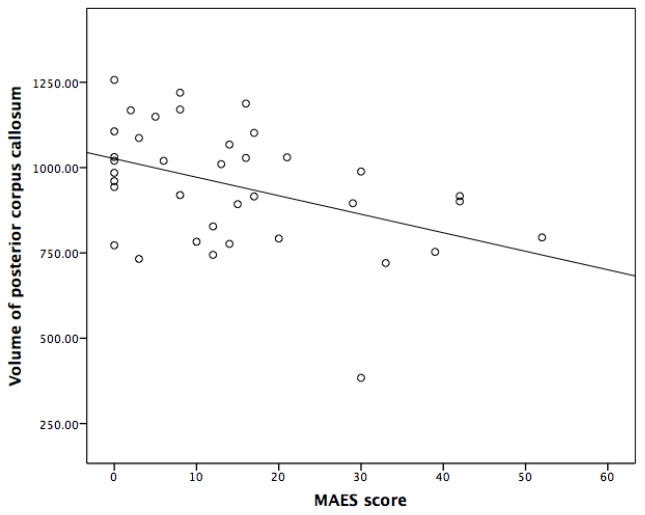
**Correlation between the Modified Apathy Evaluation Scale (MAES) score and the volume of posterior corpus callosum in AD patients.**

**Figure 2:**
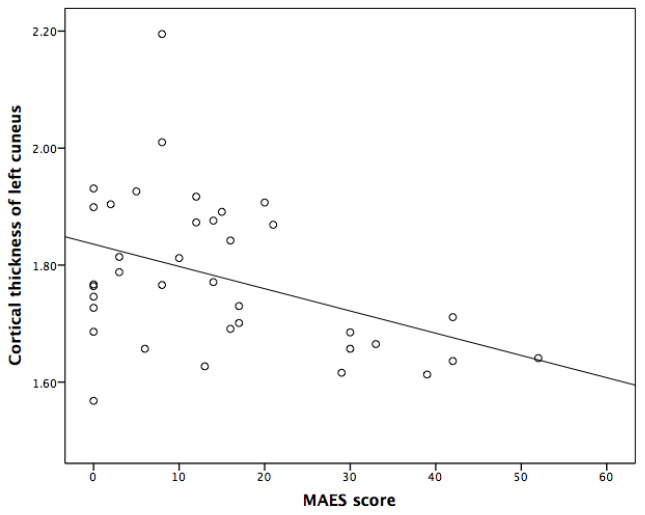
**Correlation between the Modified Apathy Evaluation Scale (MAES) score and the cortical thickness of left cuneus in AD patients.**

**Figure 3:**
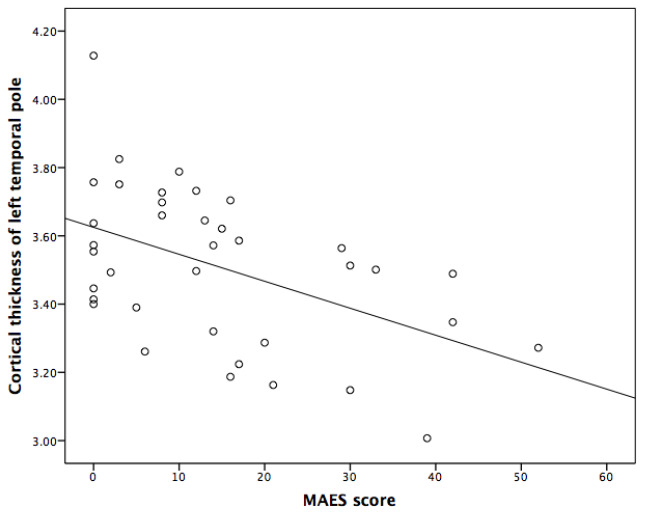
**Correlation between the Modified Apathy Evaluation Scale (MAES) score and the cortical thickness of left temporal pole in AD patients.**

Spearman correlation analyses indicated that the thicknesses of left cuneus and left temporal pole, and the volume of posterior corpus callosum were significantly and negatively correlated with MAES score (P<0.05) (Tables 3, 4 and Figures 1–3).

**Table 3:** Spearman correlation analyses between the cortical thickness and MAES score.

**Cortical thickness of left hemisphere**	**Coefficient (P value)**	**Cortical thickness of right hemisphere**	**Coefficient (P value)**
Caudal anterior cingulate	-0.126(0.457)	Caudal anterior cingulate	-0.068(0.690)
Caudal middle frontal cortex	-0.260(0.121)	Caudal middle frontal cortex	-0.161(0.341)
Cuneus	-0.362(0.028*)	Cuneus	-0.203(0.229)
Entorhinal cortex	-0.002(0.992)	Entorhinal cortex	0.000(1.000)
Fusiform	-0.141(0.404)	Fusiform	-0.138(0.416)
Inferior parietal cortex	-0.074(0.664)	Inferior parietal cortex	-0.205(0.223)
Inferior temporal cortex	-0.098(0.564)	Inferior temporal cortex	-0.135(0.426)
Isthmus cingulate	0.008(0.964)	Isthmus cingulate	-0.052(0.760)
Occipital cortex	-0.157(0.354)	Lateral occipital cortex	-0.121(0.474)
Orbitofrontal cortex	-0.127(0.453)	Lateral orbitofrontal cortex	-0.063(0.712)
Gyrus lingualis	-0.272(0.103)	Gyrus lingualis	-0.212(0.209)
Medial orbitofrontal cortex	0.106(0.532)	Medial orbitofrontal cortex	0.078(0.648)
Middle temporal cortex	-0.013(0.939)	Middle temporal cortex	-0.020(0.905)
Parahippocampi	-0.057(0.738)	Parahippocampi	-0.009(0.956)
Paracentral cortex	-0.212(0.208)	Paracentral cortex	-0.088(0.603)
Pars opercularis	-0.229(0.173)	Pars opercularis	0.092(0.590)
Pars orbitalis	-0.070(0.682)	Pars orbitalis	0.142(0.041)
Pars triangularis	0.033(0.848)	Pars triangularis	0.107(0.528)
Pericalcarine cortex	-0.218(0.195)	Pericalcarine cortex	0.004(0.984)
Postcentral cortex	-0.064(0.707)	Postcentral cortex	-0.196(0.244)
Posterior cingulate	-0.134(0.429)	Posterior cingulate	0.011(0.950)
Precentral cortex	-0.151(0.371)	Precentral cortex	-0.043(0.803)
Precuneus	-0.145(0.392)	Precuneus	-0.059(0.730)
Rostral anterior cingulate	-0.029(0.867)	Rostral anterior cingulate	-0.155(0.360)
Rostral middle frontal cortex	0.158(0.350)	Rostral middle frontal cortex	0.026(0.880)
Superior frontal cortex	-0.177(0.294)	Superior frontal cortex	-0.082(0.631)
Superior parietal cortex	-0.247(0.141)	Superior parietal cortex	-0.192(0.256)
Superior temporal cortex	-0.129(0.447)	Superior temporal cortex	-0.032(0.852)
Supramarginal cortex	-0.168(0.319)	Supramarginal cortex	-0.096(0.572)
Frontal pole	0.140(0.409)	Frontal pole	-0.067(0.695)
Temporal pole	-0.451(0.005*)	Temporal pole	0.048(0.777)
Transverse temporal cortex	-0.209(0.214)	Transverse temporal cortex	0.054(0.753)
Insula	-0.153(0.365)	Insula	-0.188(0.266)
Mean	-0.214(0.204)	Mean	-0.157(0.355)

*: P<0.05.

**Table 4:** Spearman correlation analyses between the cortical volume and MAES score.

**The volume of grey matter**	**Coefficient (P value)**
**Left hemisphere**	
Hippocampus	-0.187(0.268)
Amygdala	-0.156(0.356)
Accumbens area	-0.050(0.767)
Thalamus Proper	-0.041(0.811)
Caudate	-0.292(0.079)
Putamen	-0.163(0.334)
Pallidum	-0.014(0.932)
**Right hemisphere**	
Hippocampus	-0.041 (0.800)
Amygdala	-0.132(0.417)
Accumbens area	-0.016(0.925)
Thalamus Proper	-0.044(0.798)
Caudate	-0.237(0.158)
Putamen	-0.275(0.100)
Pallidum	-0.183(0.258)
Posterior corpus callosum	-0.384 (0.019*)
Middle posterior corpus callosum	-0.057(0.740)
Central corpus callosum	-0.061(0.722)
Middle anterior corpus callosum	-0.085(0.615)
Anterior corpus callosum	-0.255(0.128)
Left cortex	-0.228(0.175)
Right cortex	-0.214(0.203)
Total cortex	-0.232(0.168)
Sub cortical gray matter	-0.231(0.168)
Total Gray matter	-0.230(0.170)

*: P<0.05

Incorporation of confounding factors, such as age, disease duration, the scores of MMSE and NPI, further multiple linear analyses revealed that the score of MAES was independently correlated with the thickness of left temporal pole and the volume of posterior corpus callosum, which could explain 24.2% and 11.1% variance of the thickness and volume of above brain structures, respectively (Table 5).

**Table 5:** Multiple linear regression analyses between the cortical thickness/volume and related factors.

**Factors**	**Included variables**	**Excluded variables**	**β**	**P**	**ΔR2**	**Adjusted R2**
Left cuneus	MMSE	Age, disease duration, NPI and MAES scores	0.422	0.016*	0.178	0.151
Left temporal pole	MEAS	Age, disease duration, NPI and MMSE scores	-0.516	0.002*	0.267	0.242
Posterior corpus callosum	MEAS	Age, disease duration, NPI and MMSE scores	-0.373	0.035*	0.139	0.111

*: P<0.05.

## DISCUSSIONS

In this study, the frequency of apathy in AD patients reached up to 48.2%. A meta analyses covering 25 studies reported a prevalence of apathy in AD ranging from 19% to 88%, with a mean prevalence of 49%. The heterogeneity of apathy frequency across studies might depend upon the apathy scale used, disease severity and education level [8]. However, all these studies demonstrated that apathy was very common in AD patients.

We didn’t find significant differences in age, gender, disease duration and education level between the AD-A and AD-NA groups. However, the score of MMSE scale was significantly lowered in AD-A group, and was negatively correlated with MAES score (P<0.05). Some scholars speculated that apathy might be in the chain of causality leading to symptomatic AD by virtue of its effect on the brain, such as activations of the neuroendocrine axis [9]. In this context, the symptom of apathy might reflect an underlying pathology or brain state that was causally linked to the development of pathology and related cognitive symptoms of AD [9].

Besides apathy, depression, anxiety and other neuropsychiatric symptoms (NPSs) are very common in AD. NPSs bring about heavy care burdens to the family members of AD patients, and thus most patients have to be hospitalized. NPSs typically emerge in three phases, with irritability, depression and nighttime behavior changes in phases 1, anxiety, appetite changes, agitation and apathy in phases 2, and elation, hallucinations, delusions and disinhibition in phases 3 [10]. AD pathology was found in the key locations of the limbic system, including the amygdala, basal forebrain, hypothalamus and brainstem. For example, neurofibrillary tangles were abundant in the amygdala, as well as basal nucleus of Meynert, locus coeruleus, substantia nigra, dorsal raphe nucleus and hypothalamus of AD patients. These brain regions were important for mediating many aspects of emotional experience and regulating appetite, sleep and circadian rhythms [11]. Thus, the common pathological changes might explain the substantial overlap and close relation among these symptoms.

In this investigation, it was found that apathy significantly compromised the ADL of AD patients. Other studies also observed that apathy caused impairment in the functional activities of AD patients [12]. Therefore, clinicians should pay great attention to the early identification and intervention of apathy and thus improve the quality of life of AD patients.

The ε4 allele of ApoE is the most robust risk gene for the late onset AD. Individuals carrying ε4 allele of ApoE had significantly increased risk of AD compared with those carrying ε3 allele. By contrast, ε2 allele decreased the risk of AD [13, 14]. However, there is no evidence indicating the direct association between ApoE genotype and apathy of AD. In this investigation, the frequency of all ApoE genotypes, including ε2/ ε2, ε2/ ε3, ε2/ ε4, ε3/ ε3, ε3/ ε4, and ε4/ ε4, was not significantly different between the AD-A and AD-NA groups (P>0.05), which demonstrated that ApoE genotype had no influence on apathy in AD patients. Previous study showed the evidence that both APOE ε4 and apathy increased the risk of AD, and the hazard of developing AD was almost eleven times higher for ε4 carriers with apathy [15]. In another study, apathy and APOE ε4 were both associated with the reduced levels of brain-derived neurotrophic factor (BDNF) in AD patients, which was considered as a pathogenic event of AD [16]. However, a systematic review and meta-analysis including 53 studies found no association between apathy and APOE ε4 [17], which was consistent with our results. We suspected that ApoE ε4 and apathy might share some common pathogenic mechanisms underlying the development of AD, such as reduced BDNF level, but directly related to each other.

In this investigation, we found that the atrophy of temporal pole presented by MRI was independently associated with apathy in AD patients. A previous study reported that the decreased thickness of inferior temporal cortex was associated with more severe apathy over time after adjusting for multiple covariates, such as sex, age, APOE genotype, premorbid intelligence, memory performance, processing speed, antidepressant use and AD duration [18]. Another cross-sectional study recruiting 46 patients with probable AD showed the medial temporal atrophy was significantly associated with apathy (odds ratio =1.605, adjusted P = 0.042) [19]. As far as we know, this is the first investigation reporting the association between the temporal pole atrophy and apathy in AD patients. Temporal pole is the part of the limbic system, involves in detecting, integrating and filtering relevant emotional, sensory and autonomic information. It can help the target brain area to generate behavioral responses to significant stimuli and participates in the process of awakening, reaction to threat and rewarding. Thus, we suspect that the atrophy of the left temporal pole may play a role on the apathy with AD.

In this study, it was also found the atrophy of posterior corpus callosum presented by MRI was independently associated with apathy in AD patients. In a previous study performed on 37 patients with moderate to severe AD in nursing home, the results displayed that the bilateral damage of the corpus callosum detected by tensor imaging (DTI) of MRI was associated with the severity of apathy (cluster size 2435, p < 0.0005, family-wise-error-corrected) [20]. In another DTI study, fractional anisotropy (FA) value in the genu of corpus callosum in AD patients with apathy was significantly decreased than those without apathy; additionally, FA values of the genu, body and splenium of corpus callosum were negatively correlated with the severity of apathy in the apathetic AD patients [21], indicating a close association between the extensive white matter damage in corpus callosum and apathy. However, this is the first report that the atrophy of temporal pole is associated with apathy in AD patients. Corpus callosum is connected with the broad regions of cortex and provides a link between cortical with subcortical structures, hence, damage to the integrity of corpus callosum and corresponding regions (including temporal cortex) might slow down the initiation, prolong the reaction time on task, and eventually cause apathy in AD patients [21].

This research indicated that the atrophy of the above two brain regions, the left temporal pole and posterior corpus callosum, were both related to apathy in AD patients. Cortical atrophy presented by MRI might be due to the degeneration or loss of neurons and glial cells. However, the pathological changes in above-mentioned brain regions that are exactly correlated to apathy of AD need further explorations through brain autopsy.

Anterior cingulate (ACC) or orbitofrontal cortex (OFC) were previously reported in AD patients. It was observed that apathy was significantly related to the atrophy of ACC and OFC presented by MRI [7, 18, 22- 24]. ACC and OFC are all involved in evaluating actions and outcomes through efferences to the basolateral amygdala and nucleus accumbens. Interestingly, amygdala and nucleus accumben are all connected with dorsolateral prefrontal cortex (DLPFC), and accordingly cause the deficits in decision-making and response to initiation. Autopsy study also supported this view. It was reported that tau in small clusters within the right ACC was associated with apathy in AD patients, and this phenomenon was particularly pronounced in the individuals with more amyloid [25], implying that atrophy of ACC might be resulted from AD pathology and consequently related to apathy in AD patients. In addition to ACC or OFC, it was reported that the neuroimaging change of other brain areas, including anterior insula [22], internal capsule [20], posterior cingulate cortex, adjacent lateral cortex, bank of superior temporal sulcus [26]**,** medial frontal cortex, pars triangularis and supplementary motor area [27], were also associated with apathy in AD patients. However, the related autopsy data is barely reported.

The results from different studies were not consistent. These discrepancies might be due to the differences in the severity of AD or the methodology used. For example, in this study, we included the score of MMSE scale as a confound factor to eliminate the influence caused by cognitive performance, whereas cognitive performance was not controlled in most of the previous studies [20]. Secondly, apathy is characterized by the loss of or diminished motivation in goal-directed behavior, cognitive activity or emotion. A previous study displayed an association between the lesion of orbital-medial prefrontal cortex and emotional blunting, and between the lesion of the medial prefrontal cortex and deficit of thinking [28]. Another study showed the evidence indicating the anatomical distinction among the different components of apathy [20]. Therefore, each individual might have different type of apathy with different brain structures affected. Hence, the heterogeneity of the population studied might lead to the discrepancy of the results. Accordingly, further investigations distinguishing different subtypes of apathy are urgently needed.

This investigation has limitations. Firstly, we did not consider contextual factors that might impact motivation, such as whether AD patients were provided with the activities that might interest them, and whether the background of each patient was enriched to avoid social isolation. Secondly, there was a lack of follow up evaluation, which was very important for understanding the brain structural changes in MRI with time extending in patients with AD-A. Thirdly, the data from normal people cohort will be collected because it is very helpful for identifying the difference among normal subjects, AD-A group and AD-NA group.

In summary, apathy is a very common symptom of AD. It is positively and significantly correlated with cognitive decline and neuropsychiatric symptoms, and significantly compromises the quality of life for AD patients. Atrophy of left temporal pole and posterior corpus callosum presented in MRI may be positively correlated with apathy of AD. The novel findings from this investigation may provide new insights into the clinical features and neuroimaging mechanisms potentially underlying the apathy of AD.

## MATRIALS AND METHODS

### Subjects

This is a cross-sectional study. A total of 137 AD patients were consecutively recruited from April 2014 to April 2017. All patients were diagnosed with typical phenotype of AD according to the International Working Group-2 criteria [29]. The exclusion criteria were as follows: the presence of neurological disorders that might affect cognition besides AD, including cerebrovascular diseases, dementia with Lewy bodies, frontotemporal dementia, corticobasal degeneration, Parkinson’s disease, multiple sclerosis, epilepsy, etc; the presence of depression or other major mental health disorders; the presence of conditions that might interfere with the completion of clinical assessments and MRI.

Among 137 AD patients, 52 cases (37.2%) were male, ages were 45-93 years old with an average of 68; 85 cases (62.5%) were female and ages were 42-88 years old with an average of 68.

All AD patients were evaluated by Modified Apathy Evaluation Scale (MAES). MAES [30] is a modified version of Apathy Evaluation Scale (AES) [31], which is an abridged version of an apathy scale designed by Robert Mann. In the evaluation, an examiner read 14 questions for each patient, and the patient chose the level for each question among “not at all”, “slightly”, “some” and “a lot”. Total score ranges from 0 to 42 point (s), and the higher score indicates more severe apathy. MAES scale was first recommended for apathy assessment in patients with Parkinson’s disease by Movement Disorders Society [32] and its sensitivity and specificity were reported as 66% and 100%, respectively in Parkinson’s disease patients, by using a cut-off value of 14 points [30]. It was also found that the intra- and interrater reliability were very high [9]. In this investigation, patients with MAES score >14 and ≤14 were divided into AD with apathy (AD-A) group and AD with no apathy (AD-NA) group, respectively. The mean score of AD-A group was significantly higher than that of AD-NA group [26.5(18.8~37.0) vs. 5.0(0~9.0), P=0.000].

Patients’ demographic information, including gender, age, age onset, disease duration and educational level, et al, was collected. Disease duration was based on the retrospective clinical information of the illness timeline.

### Assessments of clinical symptoms

Global cognitive function was assessed by Mini-Mental State Examination (MMSE) scale. Neuropsychiatric symptoms were evaluated by Neuropsychiatric Symptom Inventory (NPI) and quality of life was rated by Activities of Daily Living (ADL) scale.

### Detection of Apolipoprotein E (ApoE) genotypes

In 137 patients, 74 patients simultaneously participated in an AD genetic study and were detected ApoE genotypes, including ε2/ ε2, ε2/ε3, ε2/ ε4, ε3/ ε3, ε3/ ε4, and ε4/ ε4. Blood from each patient was taken under fasting condition and detected ApoE genotype through Real Time Fluorescence Quantitative Polymerase Chain Reaction by using nucleic acid detection reagents (Youzhiyou company, Wuhan, China) in clinical laboratory of Beijing Tiantan Hospital.

### Images collection and processing

3.0T MRI system from the Siemens (Siemens, Germany) was used for the image study. After routine three-plane positioning, the T1-weighted three-dimensional magnetization gradient echo sequence was used to get transverse brain images. The scanning range was from the cranial dome to the foramen magnum. The parameters were as followed: TR was 2300 milliseconds, TE was 2.3 milliseconds, TI was 900 milliseconds, the scanning field was 240 mm x 240 mm, the matrix was 256 x 256, the layer thickness was 1 mm and the interlayer spacing was 1 mm.

Two experts, who had 10-20 years’ experience for neuroimage reading and had no idea about the clinical information of the patients were responsible for reading and evaluating images to exclude other neurological diseases. The studies were conducted independently and consensus agreements were reached by discussions if there were disagreements.

Cortical reconstruction and volumetric segmentation were performed with the Freesurfer image analysis suite, which was freely available for download online (http://surfer.nmr.mgh.harvard.edu/). The technical details of these procedures were described in prior publications [33, 34]. Briefly, this processing included motion correction and averaging [33] of multiple volumetric T1 weighted images (when more than one were available), removal of non-brain tissue by using a hybrid watershed/surface deformation procedure [35], automated Talairach transformation, segmentation of the subcortical white matter and deep gray matter volumetric structures (including hippocampus, amygdala, caudate, putamen and ventricles) [36], intensity normalization [37], tessellation of the gray matter and white matter boundary, automated topology correction [38], and surface deformation following intensity gradients to optimally place the gray matter/white matter and gray matter/cerebrospinal fluid (CSF) borders at the location where the greatest shift in intensity defined the transition to the other tissue class [39].

Once the cortical models were completed, a number of deformable procedures were performed, including surface inflation [40], registration to a spherical atlas which was based on individual cortical folding patterns to match cortical geometry across subjects [40], parcellation of the cerebral cortex into units with respect to gyral and sulcal structure [41], and creation of a variety of surface based data, including maps of curvature and sulcal depth. This method used both intensity and continuity information from the entire three dimensional MRI volume in segmentation and deformation procedures to produce representations of cortical thickness, calculated as the closest distance from the gray matter/white matter boundary to the gray matter/CSF boundary at each vertex on the tessellated surface [42]. The maps were created by using spatial intensity gradients across tissue classes and were therefore not simply reliant on absolute signal intensity. The maps produced were not restricted to the voxel resolution of the original data, thus were capable of detecting submillimeter differences between groups. Procedures for the measurement of cortical thickness have been validated against histological analysis [43] and manual measurements [44]. Freesurfer morphometric procedures have been demonstrated to show good test-retest reliability across scanner manufacturers and across field strengths [45]. To extract reliable thickness and volume estimates, images where automatically processed with the longitudinal stream [34] in FreeSurfer. Specifically, an unbiased within-subject template space and image was created by using robust, inverse consistent registration [33]. Several processing steps, such as skull stripping, Talairach transforms, atlas registration as well as spherical surface maps and parcellations are then initialized with common information from the within-subject template, significantly increasing reliability and statistical power [34].

### Data analyses

Statistical analyses were performed with SPSS Statistics 20.0 (IBM Corporation, NY, USA).

Continuous variables were presented as mean ± standard deviation and compared by 2-tailed t test if they were normally distributed, and presented as median (quartile) and compared by nonparametric test if they were not normally distributed. Discrete variables were compared by Chi square test.

Demographic information, cognitive function, neuropsychiatric symptoms, quality of life and ApoE genotypes (ε2/ ε2, ε2/ε3, ε2/ ε4, ε3/ ε3, ε3/ ε4, and ε4/ ε4)were compared between AD-A and AD-NA groups. We have calculated the sample size before starting to recruit patients. Presuming the effect size = 0.5, *α* = 0.5, power (1-*β*) = 0.8, the sample size of each group should be no less than 64. The patients recruited were 67 cases in AD-A group and 71cases in AD-NA group, both met the demand of the sample size. Bonferroni correction was performed and the corrected P value was significant when it was less than 0.007 (0.05/7=0.007).

Due to MAES score is ordinal data, Spearman correlation analyses and further multiple linear analyses were performed between the cortical thickness or volume and related factors, including age, disease duration, the scores of MMSE, NPI and MAES. Presuming the effect size = 0.3, *α* = 0.5, power (1-*β*) = 0.8, the sample size should be no less than 82 cases. The total patients of this study were 137 cases, which met the demand of the sample size. The difference was considered to be significant when the P value was less than 0.05.

## References

[1] Hwang TJ, Masterman DL, Ortiz F, Fairbanks LA, Cummings JL. Mild cognitive impairment is associated with characteristic neuropsychiatric symptoms. Alzheimer Dis Assoc Disord. 2004; 18:17–21. 10.1097/00002093-200401000-00004 15195459

[2] Mega MS, Cummings JL, Fiorello T, Gornbein J. The spectrum of behavioral changes in Alzheimer’s disease. Neurology. 1996; 46:130–35. 10.1212/wnl.46.1.130 8559361

[3] Onyike CU, Sheppard JM, Tschanz JT, Norton MC, Green RC, Steinberg M, Welsh-Bohmer KA, Breitner JC, Lyketsos CG. Epidemiology of apathy in older adults: the cache county study. Am J Geriatr Psychiatry. 2007; 15:365–75. 10.1097/01.JGP.0000235689.42910.0d 17463187

[4] Turró-Garriga O, López-Pousa S, Vilalta-Franch J, Turón-Estrada A, Pericot-Nierga I, Lozano-Gallego M, Hernández-Ferràndiz M, Soler-Cors O, Planas-Pujol X, Monserrat-Vila S, Garre-Olmo J. [A longitudinal study of apathy in patients with Alzheimer’s disease]. Rev Neurol. 2009; 48:7–13. 19145559

[5] Robert PH, Berr C, Volteau M, Bertogliati C, Benoit M, Sarazin M, Legrain S, Dubois B, and PréAL study. Apathy in patients with mild cognitive impairment and the risk of developing dementia of Alzheimer’s disease: a one-year follow-up study. Clin Neurol Neurosurg. 2006; 108:733–36. 10.1016/j.clineuro.2006.02.003 16567037

[6] Li XL, Hu N, Tan MS, Yu JT, Tan L. Behavioral and psychological symptoms in Alzheimer’s disease. BioMed Res Int. 2014; 2014:927804. 10.1155/2014/927804 25133184 PMC4123596

[7] Theleritis C, Politis A, Siarkos K, Lyketsos CG. A review of neuroimaging findings of apathy in Alzheimer’s disease. Int Psychogeriatr. 2014; 26:195–207. 10.1017/S1041610213001725 24135083 PMC4086515

[8] Zhao QF, Tan L, Wang HF, Jiang T, Tan MS, Tan L, Xu W, Li JQ, Wang J, Lai TJ, Yu JT. Corrigendum to: “the prevalence of neuropsychiatric symptoms in Alzheimer’s disease: systematic review and meta-analysis” [J. Affect. Disord. 190 (2016) 264-271]. J Affect Disord. 2016; 206:8. 10.1016/j.jad.2016.04.054 27455352

[9] Geda YE, Schneider LS, Gitlin LN, Miller DS, Smith GS, Bell J, Evans J, Lee M, Porsteinsson A, Lanctôt KL, Rosenberg PB, Sultzer DL, Francis PT, et al, and Neuropsychiatric Syndromes Professional Interest Area of ISTAART. Neuropsychiatric symptoms in Alzheimer’s disease: past progress and anticipation of the future. Alzheimers Dement. 2013; 9:602–08. 10.1016/j.jalz.2012.12.001 23562430 PMC3766403

[10] Masters MC, Morris JC, Roe CM. “noncognitive” symptoms of early Alzheimer disease: a longitudinal analysis. Neurology. 2015; 84:617–22. 10.1212/WNL.0000000000001238 25589671 PMC4335988

[11] Lanctôt KL, Amatniek J, Ancoli-Israel S, Arnold SE, Ballard C, Cohen-Mansfield J, Ismail Z, Lyketsos C, Miller DS, Musiek E, Osorio RS, Rosenberg PB, Satlin A, et al. Neuropsychiatric signs and symptoms of Alzheimer’s disease: new treatment paradigms. Alzheimers Dement (N Y). 2017; 3:440–49. 10.1016/j.trci.2017.07.001 29067350 PMC5651439

[12] Lechowski L, Benoit M, Chassagne P, Vedel I, Tortrat D, Teillet L, Vellas B. Persistent apathy in Alzheimer’s disease as an independent factor of rapid functional decline: the REAL longitudinal cohort study. Int J Geriatr Psychiatry. 2009; 24:341–46. 10.1002/gps.2125 18814198

[13] Bettens K, Sleegers K, Van Broeckhoven C. Genetic insights in Alzheimer’s disease. Lancet Neurol. 2013; 12:92–104. 10.1016/S1474-4422(12)70259-4 23237904

[14] Liu CC, Liu CC, Kanekiyo T, Xu H, Bu G. Apolipoprotein E and Alzheimer disease: risk, mechanisms and therapy. Nat Rev Neurol. 2013; 9:106–18. 10.1038/nrneurol.2012.263 23296339 PMC3726719

[15] Burke SL, Maramaldi P, Cadet T, Kukull W. Neuropsychiatric symptoms and apolipoprotein E: associations with eventual Alzheimer’s disease development. Arch Gerontol Geriatr. 2016; 65:231–38. 10.1016/j.archger.2016.04.006 27111252 PMC5029123

[16] Alvarez A, Aleixandre M, Linares C, Masliah E, Moessler H. Apathy and APOE4 are associated with reduced BDNF levels in Alzheimer’s disease. J Alzheimers Dis. 2014; 42:1347–55. 10.3233/JAD-140849 25024337 PMC4931817

[17] Banning LC, Ramakers IH, Deckers K, Verhey FR, Aalten P. Apolipoprotein E and affective symptoms in mild cognitive impairment and Alzheimer’s disease dementia: a systematic review and meta-analysis. Neurosci Biobehav Rev. 2019; 96:302–15. 10.1016/j.neubiorev.2018.11.020 30513312

[18] Donovan NJ, Wadsworth LP, Lorius N, Locascio JJ, Rentz DM, Johnson KA, Sperling RA, Marshall GA, and Alzheimer Disease Neuroimaging Initiative. Regional cortical thinning predicts worsening apathy and hallucinations across the Alzheimer disease spectrum. Am J Geriatr Psychiatry. 2014; 22:1168–79. 10.1016/j.jagp.2013.03.006 23890751 PMC3960369

[19] García-Alberca JM, Florido M, Cáceres M, Sánchez-Toro A, Lara JP, García-Casares N. Medial temporal lobe atrophy is independently associated with behavioural and psychological symptoms in Alzheimer's disease. Psychogeriatrics. 2019; 19:46–54. 10.1111/psyg.12363 30084177

[20] Agüera-Ortiz L, Hernandez-Tamames JA, Martinez-Martin P, Cruz-Orduña I, Pajares G, López-Alvarez J, Osorio RS, Sanz M, Olazarán J. Structural correlates of apathy in Alzheimer’s disease: a multimodal MRI study. Int J Geriatr Psychiatry. 2017; 32:922–30. 10.1002/gps.4548 27428560

[21] Hahn C, Lim HK, Won WY, Ahn KJ, Jung WS, Lee CU. Apathy and white matter integrity in Alzheimer’s disease: a whole brain analysis with tract-based spatial statistics. PLoS One. 2013; 8:e53493. 10.1371/journal.pone.0053493 23301077 PMC3536751

[22] Moon Y, Moon WJ, Kim H, Han SH. Regional atrophy of the insular cortex is associated with neuropsychiatric symptoms in Alzheimer’s disease patients. Eur Neurol. 2014; 71:223–29. 10.1159/000356343 24480815

[23] Tunnard C, Whitehead D, Hurt C, Wahlund LO, Mecocci P, Tsolaki M, Vellas B, Spenger C, Kłoszewska I, Soininen H, Lovestone S, Simmons A, and AddNeuroMed Consortium. Apathy and cortical atrophy in Alzheimer’s disease. Int J Geriatr Psychiatry. 2011; 26:741–48. 10.1002/gps.2603 20872914

[24] Bruen PD, McGeown WJ, Shanks MF, Venneri A. Neuroanatomical correlates of neuropsychiatric symptoms in Alzheimer’s disease. Brain. 2008; 131:2455–63. 10.1093/brain/awn151 18669506

[25] Marshall GA, Gatchel JR, Donovan NJ, Muniz MC, Schultz AP, Becker JA, Chhatwal JP, Hanseeuw BJ, Papp KV, Amariglio RE, Rentz DM, Sperling RA, Johnson KA. Regional tau correlates of instrumental activities of daily living and apathy in mild cognitive impairment and Alzheimer’s disease dementia. J Alzheimers Dis. 2019; 67:757–68. 10.3233/JAD-170578 30689584 PMC6352915

[26] Huey ED, Lee S, Cheran G, Grafman J, Devanand DP, and Alzheimer’s Disease Neuroimaging Initiative. Brain regions involved in arousal and reward processing are associated with apathy in Alzheimer’s disease and frontotemporal dementia. J Alzheimers Dis. 2017; 55:551–58. 10.3233/JAD-160107 27802220

[27] Kos C, van Tol MJ, Marsman JB, Knegtering H, Aleman A. Neural correlates of apathy in patients with neurodegenerative disorders, acquired brain injury, and psychiatric disorders. Neurosci Biobehav Rev. 2016; 69:381–401. 10.1016/j.neubiorev.2016.08.012 27527825

[28] Levy R, Dubois B. Apathy and the functional anatomy of the prefrontal cortex-basal ganglia circuits. Cereb Cortex. 2006; 16:916–28. 10.1093/cercor/bhj043 16207933

[29] Dubois B, Feldman HH, Jacova C, Hampel H, Molinuevo JL, Blennow K, DeKosky ST, Gauthier S, Selkoe D, Bateman R, Cappa S, Crutch S, Engelborghs S, et al. Advancing research diagnostic criteria for Alzheimer’s disease: the IWG-2 criteria. Lancet Neurol. 2014; 13:614–29. 10.1016/S1474-4422(14)70090-0 24849862

[30] Starkstein SE, Mayberg HS, Preziosi TJ, Andrezejewski P, Leiguarda R, Robinson RG. Reliability, validity, and clinical correlates of apathy in Parkinson’s disease. J Neuropsychiatry Clin Neurosci. 1992; 4:134–39. 10.1176/jnp.4.2.134 1627973

[31] Marin RS. Differential diagnosis and classification of apathy. Am J Psychiatry. 1990; 147:22–30. 10.1176/ajp.147.1.22 2403472

[32] Starkstein SE. Apathy in Parkinson’s disease: diagnostic and etiological dilemmas. Mov Disord. 2012; 27:174–78. 10.1002/mds.24061 22237755

[33] Reuter M, Rosas HD, Fischl B. Highly accurate inverse consistent registration: a robust approach. Neuroimage. 2010; 53:1181–96. 10.1016/j.neuroimage.2010.07.020 20637289 PMC2946852

[34] Reuter M, Schmansky NJ, Rosas HD, Fischl B. Within-subject template estimation for unbiased longitudinal image analysis. Neuroimage. 2012; 61:1402–18. 10.1016/j.neuroimage.2012.02.084 22430496 PMC3389460

[35] Ségonne F, Dale AM, Busa E, Glessner M, Salat D, Hahn HK, Fischl B. A hybrid approach to the skull stripping problem in MRI. Neuroimage. 2004; 22:1060–75. 10.1016/j.neuroimage.2004.03.032 15219578

[36] Fischl B, Salat DH, van der Kouwe AJ, Makris N, Ségonne F, Quinn BT, Dale AM. Sequence-independent segmentation of magnetic resonance images. Neuroimage. 2004 (Suppl 1); 23:S69–84. 10.1016/j.neuroimage.2004.07.016 15501102

[37] Sled JG, Zijdenbos AP, Evans AC. A nonparametric method for automatic correction of intensity nonuniformity in MRI data. IEEE Trans Med Imaging. 1998; 17:87–97. 10.1109/42.668698 9617910

[38] Ségonne F, Pacheco J, Fischl B. Geometrically accurate topology-correction of cortical surfaces using nonseparating loops. IEEE Trans Med Imaging. 2007; 26:518–29. 10.1109/TMI.2006.887364 17427739

[39] Dale AM, Fischl B, Sereno MI. Cortical surface-based analysis. I. Segmentation and surface reconstruction. Neuroimage. 1999; 9:179–94. 10.1006/nimg.1998.0395 9931268

[40] Fischl B, Sereno MI, Dale AM. Cortical surface-based analysis. II: Inflation, flattening, and a surface-based coordinate system. Neuroimage. 1999; 9:195–207. 10.1006/nimg.1998.0396 9931269

[41] Desikan RS, Ségonne F, Fischl B, Quinn BT, Dickerson BC, Blacker D, Buckner RL, Dale AM, Maguire RP, Hyman BT, Albert MS, Killiany RJ. An automated labeling system for subdividing the human cerebral cortex on MRI scans into gyral based regions of interest. Neuroimage. 2006; 31:968–80. 10.1016/j.neuroimage.2006.01.021 16530430

[42] Fischl B, Dale AM. Measuring the thickness of the human cerebral cortex from magnetic resonance images. Proc Natl Acad Sci USA. 2000; 97:11050–55. 10.1073/pnas.200033797 10984517 PMC27146

[43] Rosas HD, Liu AK, Hersch S, Glessner M, Ferrante RJ, Salat DH, van der Kouwe A, Jenkins BG, Dale AM, Fischl B. Regional and progressive thinning of the cortical ribbon in Huntington’s disease. Neurology. 2002; 58:695–701. 10.1212/wnl.58.5.695 11889230

[44] Salat DH, Buckner RL, Snyder AZ, Greve DN, Desikan RS, Busa E, Morris JC, Dale AM, Fischl B. Thinning of the cerebral cortex in aging. Cereb Cortex. 2004; 14:721–30. 10.1093/cercor/bhh032 15054051

[45] Han X, Jovicich J, Salat D, van der Kouwe A, Quinn B, Czanner S, Busa E, Pacheco J, Albert M, Killiany R, Maguire P, Rosas D, Makris N, et al. Reliability of MRI-derived measurements of human cerebral cortical thickness: the effects of field strength, scanner upgrade and manufacturer. Neuroimage. 2006; 32:180–94. 10.1016/j.neuroimage.2006.02.051 16651008

